# Maternal personality disorder symptoms in primary health care: associations with mother–toddler interactions at one-year follow-up

**DOI:** 10.1186/s12888-018-1789-5

**Published:** 2018-06-18

**Authors:** Magnhild Singstad Høivik, Stian Lydersen, Ingunn Ranøyen, Turid Suzanne Berg-Nielsen

**Affiliations:** 1 Department of Mental Health, Faculty of Medicine and Health Sciences, the Norwegian University of Science and Technology (NTNU), N-7491 Trondheim, Norway; 2 Regional Centre for Child and Youth Mental Health and Child Welfare – Central Norway, Faculty of Medicine and Health Sciences, The Norwegian University of Science and Technology (NTNU), Trondheim, Norway; 3 Division of Psychiatry, St Olavs Hospital, Trondheim University Hospital, Trondheim, Norway; 4 The Centre for Child and Adolescent Mental Health, Eastern and Southern Norway, Oslo, Norway

**Keywords:** Personality disorder symptoms, Mother–toddler interactions, Longitudinal study

## Abstract

**Background:**

Research is scarce on how mothers’ symptoms of personality disorders are linked to the mother-toddler relationship. In this study we have explored the extent to which these symptoms are associated with mutual mother-toddler interactions assessed 1 year after the initial assessment.

**Methods:**

Mothers and their 0–24-month-old children (*n* = 112) were recruited by nurses at well-baby clinics due to either self-reported or observed mother–toddler interaction problems. At inclusion (T1), mothers filled out the DSM-IV and ICD-10 Personality Questionnaire (DIP-Q), which measures symptoms of ten personality disorders. A year later (T2), mother-toddler interactions were video-recorded and coded using a standardised observation measure, the Emotional Availability Scales.

**Results:**

Only maternal schizotypal personality disorder symptoms predicted both the mothers’ and the toddlers’ interactional styles. Mothers with schizotypal personality symptoms appeared less sensitive, less structuring and more intrusive in their interactions with their toddlers, while mothers’ borderline personality disorder symptoms were associated with increased hostility. Furthermore, toddlers who had mothers with schizotypal personality symptoms were less responsive towards their mothers.

**Conclusion:**

Measured dimensionally by self-report, maternal schizotypal personality symptoms were observed to predict the interaction styles of both mothers and their toddlers in the dyad, while borderline personality disorder symptoms predicted mothers’ interactional behaviour only.

**Trial registration:**

Current Controlled Trials ISRCTN99793905, retrospectively registered. Registered on (04/08/2014).

**Electronic supplementary material:**

The online version of this article (10.1186/s12888-018-1789-5) contains supplementary material, which is available to authorized users.

## Background

How mothers’ personality disorders (PDs) affect the mother–child relationship has attracted far less attention in research than might be expected, given that chronic parental mental illness is, in general, thought to be an important predictor of maladaptive parenting [1–4]. For most PDs, other than borderline, there are knowledge gaps regarding their effect on observed mother–toddler interactions.

Generally, individuals with PD diagnoses show characteristic pervasive, inflexible and stable deviant patterns of behaviour and experiences in social relations. The DSM-5 defines PD as a failure to develop a sense of self-identity and capacity for interpersonal functioning that are adaptive in the context of the individual’s norms and expectations [5]. Furthermore, central elements of personality organisation are affected, such as the ability to flexibly regulate impulses and affect and effectively cope with stressful events [6]. Because of their apparent problems with emotional regulation and self-control, mothers with PDs might be particularly challenged when faced with a child’s negative affect or difficult temperament [7]. Accordingly, maternal symptoms of PDs putatively suggest the risk that these mothers may not be emotionally or behaviourally stable, predictable care-providers in interactions with their children. Research relying on parents’ own reports on their parenting does indeed support this assumption of associations between problematic parenting behaviours and mothers’ PDs [3, 4, 8–10]. Some families may also be faced with ‘a double risk’ of mother–toddler relationship problems because PDs are hereditary conditions, potentially causing the offspring to have challenging temperamental traits and/or negative emotionality [11].

Direct observation of interactions has been the method of choice when investigating the parent–child relationship to reduce the effect of parental recall bias or general lack of self-observation ability [12, 13]. Challenging child temperament may, however, contribute as much to shaping parenting behaviours as parent psychopathology does [14–19]. For this reason, it is imperative to consider the child’s responses to the behavioural exchanges in the interaction when coding parental sensitivity [20]. Several approaches take account of emotional aspects when coding interactions [21, 22], which are inevitably included when evaluating personality disorders in association with the parent-toddler relationship [7]. To the best of our knowledge, the small body of literature focusing on the associations between all the ten PDs and parent–toddler interactions has not covered the effect of PDs on the nonverbally and bi-directionally displayed emotional aspects of the parent–toddler dyad [23], which is the objective of the current study.

Most of the existing research on the effect of PDs on parent–child interactions has included clinical adult/child samples or parents with symptoms meeting the diagnostic criteria for a PD [7, 24, 25]. The associations between mothers’ symptoms of PDs and mother–toddler interactions are likely stronger in clinical samples because of lower levels of general functioning and more distress and co-morbid psychiatric conditions, such as multiple PD diagnoses, cognitive dysfunction or social problems [7, 24–28]. Given the close relationship with the child, less disturbed self-other representations or interpersonal functioning in relation to parental PD might also influence the dyadic interaction [24, 29]. The aim of the current study is therefore to explore the associations between parental PD symptoms and interactions with toddlers in families where parents have problems considered non-clinical in terms of prevalence of the most serious, pervasive PD conditions, even though they were recruited because of parent–child interaction problems.

### Associations between maternal PDs and parent-child interactions

The limited, but informative body of literature focusing on possible associations between mothers’ PDs and their ability to engage in *positive* connections (e.g. emotional involvement, sensitivity and responsiveness) with their infants and toddlers, have linked less maternal positive interactions with all the Cluster A (paranoid, schizoid and schizotypal) [7, 24, 25, 29], three of the four Cluster B (histrionic, antisocial, and borderline) [24, 29] and all the Cluster C (avoidant, dependent and obsessive-compulsive) [24, 25] PD spectrums in samples of normal mothers or mothers diagnosed with PDs and/or affective disorders.

The research literature focuses on *negative* parenting, such as emotional over-involvement, frightening, hostile, unpredictable, or intrusive behaviours has mainly been concerned with Cluster B PDs [30–34]. Besides parents’ inadequate capacities to cope with stress and regulate emotions effectively, the literature suggests that negative parenting is related to distorted and biased hostile attributions to the child, as well as reflective functioning impairment [13, 26, 35, 36]. However, to the best of our knowledge, until now only three studies have investigated how mothers’ diagnoses or dimensional measures within all three PD clusters/all ten PDs, are linked to these negative aspects of maternal involvement in mother-infant/toddler interactions [7, 24, 29]. In these publications, negative parenting behaviours are linked to maternal Cluster A [7, 24, 29] as well as narcissistic PD and obsessive-compulsive PD symptoms [29].

The main body of research in this field has, however, covered the effect of maternal borderline PD on parent-child interactions (for overviews of the litterature; [31, 33, 36]). This evidence links mothers’ less positive parenting and more negative parenting and all ranges of maternal borderline PD symptomatology in clinical and non-clinical samples [31, 33, 36].

How the child responds to emotional or behavioural exchanges initiated by the mother (i.e., to what degree the child shows positive affect and flexibly regulates/organises emotions and behaviours) has primarily been investigated in mothers with borderline PD diagnoses. In studies of borderline PD mothers recruited from both community and clinical settings, their infants were observed as being less responsive, more avoidant and showing increased negative affect during face-to-face dialogues [37–40].

To the best of our knowledge, only one study has investigated how symptoms of other PDs in mothers affect children’s behaviour in mother-child interactions [29]. The researchers observed lower levels of 6-year-olds’ positive interactions when parents had schizoid PD symptoms, while toddlers’ compliant, but not responsive behaviours were associated with maternal paranoid PD symptoms.

### Aims

The primary aim of the present study was to explore the possible associations between maternal PD symptoms and the following aspects of mother–toddler interactions:

- mother’s sensitivity to child’s signals,
- mother’s capacity to structure the interaction,
- mother’s non-intrusiveness,
- mother’s non-hostility
- toddler’s responsiveness and
- toddler’s involvement of mother

Since our sample was selected from a randomised controlled trial (RCT) of the effect of an intervention that provided video feedback of infant–mother interactions [41], the secondary objective was to explore the possible moderating effects of intervention on the associations between maternal PDs and mentioned aspects of mother-toddler interactions.

## Methods

### Study design

The study had a multi-site, naturalistic, longitudinal design, involving families from urban and rural samples in Norway recruited at well-baby clinics.

### Participants

During the period from March 2008 to September 2012, 152 families were recruited and accepted into the study and were given a baseline evaluation (T1). The families were recruited from well-baby units in the cities of Trondheim and Oslo and in six rural communities in eastern Norway. The participants were all biological mothers (Table 1). Inclusion criteria in the study were mothers asking for help with problems in handling their baby or toddler (applied to 50.9% of cases), or a recommendation from the well-baby nurse to receive help for mother–toddler interaction problems (49.1%), and an age of 0–24 months for the child at the time of inclusion. Mothers who had substance abuse problems, ongoing psychosis, developmental disorders or who did not have sufficient proficiency in Norwegian to answer the questionnaires were excluded. There were no child-related exclusion criteria.

**Table 1:** Sample demographic characteristics

Characteristics	*n* or mean (sd)	Percent
Children’s characteristics		
Child living with	140	
Both parents		82.9
Biological mother		15.7
Mother and stepfather		0.7
Mother and father alternately	140	0.7
Age at inclusion (months)	7.3 (5.1)	
Child’s gender	141	
Boy		49.0
Girl		51.0
Cohabitant siblings	137	
First-born child		72.0
Older siblings		28.0
Parental characteristics		
Mother on maternity leave	141	63.8
Mother in work	140	35.7
Other activity (student, unemployed, etc.)	140	0.7
Age of mother at inclusion	140 29.7 (5.6)	
Ethnic origin of mother	96	
Norwegian		82.6
Other European		6.5
African		3.3
Asian		5.4
South American		2.2
Maternal educational level at inclusion	140	
Junior high school		5.7
Senior high school		12.1
Vocational education (1–2 years)		19.3
Bachelor’s degree		25.0
Master’s degree or higher		37.9
Ongoing education, mother	130	
Yes		18.7
No		81.3
Age of father at inclusion	134 32.8 (7.0)	
Ethnic origin of father	93	
Norwegian		89.8
Other European		6.8
African		2.3
North American		1.1
Paternal educational level at inclusion	135	
Junior high school		5.3
Senior high school		17.3
Vocational education (1–2 years)		19.5
Bachelor’s degree		30.8
Master’s degree or higher		27.1
Ongoing education, father	132	
Yes		13.3
No		86.7
Earlier/ongoing psychiatric illness	140	
Mother		17.5
Father		5.6
Family monthly income, after taxes (in 1000 NKr)	135 33.9 (17.5)	
Experienced support (partner/mothers/other family/friends/professionals)	140	
Satisfied (very/a little)		90.0/9.3
Unsatisfied (very/a little)		0.7/10.0
Conflicts in close relations (partner/family/friends/colleagues)	127	
Never/hardly ever		62.6/87.1
Sometimes		4.4/29.4
Often/very often		4.0/11.4

Forty families withdrew or were excluded during the study period (for example, the mothers lost child custody, became psychotic, were hospitalised, moved away, etc.), leaving 112 families to participate in the second evaluation after 11.5 months (range 9 to 13 months) (T2) (Table 2).

**Table 2:** Sample clinical characteristics at baseline and at one-year follow-up

	Baseline									Follow up					
	All participants			Attrition group			Remaining group								
	*n*	mean	sd	*n*	mean	sd	*n*	mean	sd	*n*	mean	sd	cut off (diagnosis)	*n* (%) with symptoms over cut off	Scale range
Personality disorder symptoms															
Avoidant	122	1.93	1.95	28	2.29	2.56	94	1.82	1.85	–	–	–	≥ 4	26 (21.3)	0–8
Dependent	122	1.81	1.88	28	**2.61** ^***^	2.39	94	**1.57** ^***^	1.64	–	–	–	≥ 5	13 (10.7)	0–8
Obsessive-compulsive	122	3.88	1.74	28	4.00	1.89	94	3.84	1.70	–	–	–	≥ 4	69 (56.6)	0–11
Paranoid	122	1.34	1.60	28	**2.04** ^***^	2.03	94	**1.14** ^***^	1.40	–	–	–	≥ 5	8 (6.6)	0–16
Schizoid	122	0.73	0.97	28	0.68	0.86	94	0.75	1.00	–	–	–	≥ 4	1 (0.8)	0–8
Schizotypal	122	0.41	1.66	28	2.04	1.88	94	1.22	1.55	–	–	–	≥ 5	10 (8.2)	0–10
Antisocial	122	0.85	0.85	28	0.86	0.85	94	0.85	0.94	–	–	–	≥ 3	6 (4.9)	0–10
Borderline	122	2.48	2.15	28	**3.61** ^***^	2.69	94	**2.14** ^***^	1.85	–	–	–	≥ 5	19 (15.6)	0–7
Histrionic	122	1.25	1.22	28	1.39	1.17	94	1.21	1.24	–	–	–	≥ 5	1 (0.8)	0–9
Narcissistic	122	0.86	1.05	28	**1.29** ^**^	1.33	94	**0.73** ^**^	0.87	–	–	–	≥ 5	1 (0.8)	0–9
Impairment and distress	122	0.66	1.03	28	**0.80** ^*^	1.26	94	**0.63** ^*^	0.96	–	–	–	≥ 2	20 (16.4)	0–5
Depressive symptoms	118	12.11	8.64	24	**15.96** ^*^	11.23	94	**10.99** ^**^	7.84	85	**8.74** ^***^	7.05	–	–	0–64
EAS subscales															
Maternal sensitivity	152	22.41	5.12	42	21.48	4.94	110	22.77	5.16	110	**25.29** ^***^	3.92	–	–	7–29
Maternal structuring	152	23.26	4.50	42	22.38	4.29	110	23.60	4.55	110	**25.90** ^***^	3.39	–	–	7–29
Maternal non-hostility	152	26.01	3.58	42	**24.92** ^*^	4.18	110	**26.44** ^*^	3.24	110	**27.32** ^***^	2.50	–	–	7–29
Maternal non-intrusiveness	152	22.24	5.72	42	21.26	6.16	110	22.63	5.52	110	**25.27** ^***^	4.34	–	–	7–29
Child responsiveness	152	22.66	5.36	42	21.59	5.06	110	23.07	5.43	110	**25.70** ^***^	4.08	–	–	7–29
Child involvement	152	22.43	5.94	42	20.50	5.97	110	21.79	5.92	110	**25.11** ^***^	4.71	–	–	7–29

Maternal personality disorder symptoms measured with the DIP-Q (DSM IV and ICD-10 Personality Questionnaire)

^*^ = *p* < 0.05, ^**^ = *p* < 0.01, ^***^ = *p* < 0.001 (independent samples t-tests of characteristics in attrition compared to remaining groups in baseline sample, and paired sample t-tests comparing characteristics of remaining and follow up groups). Significant findings are shown bold

### Procedure and assessment

Since one might expect the presence of transactional patterns between mothers’ psychopathology and interactions with their toddlers, we employed a longitudinal rather than a cross-sectional design when investigating possible associations between maternal PD symptoms and mother–toddler interactions. We expected that the older the child, the unhealthier the interaction circuits [42]. Consequently, we used the subscales of a personality disorder questionnaire at baseline as predictor, while our dependent variables were aspects of the mother-toddler interactions measured 1 year later.

To make the participation as easy as possible for the families, trained research assistants with a bachelor’s degree in nursing, social work or preschool education met the families at home. The research assistants also offered to travel to visit the families if they moved out of the recruitment district in order to reduce inconveniences for the families and thereby reduce the levels of attrition.

The mothers completed self-report questionnaires addressing PD symptoms as well as socio-economic and demographic information at inclusion (T1). Approximately 1 year later (mean: 11.5 months) (T2), 30-min videos were recorded in the participants’ homes in everyday situations, e.g. whilst they were playing, feeding or nappy changing. The mothers were instructed to ‘interact with their toddler as they usually would’ and were free to choose activities and time points for videotaping. The videos were assessed according to a standardised observational method. We coded and included in the analysis 110 of 112 videos of mother–toddler interactions; two recordings were damaged. The observational measure’s behaviour dimensions constituted the four adult outcomes and the two child outcomes in the study.

#### Baseline assessment (T1)

##### Maternal personality disorder symptoms

*DSM-IV and ICD-10 Personality Questionnaire (DIP-Q)* [43]*.* The DIP-Q is a 140-item true/false self-report scale addressing personality symptoms that meet the diagnostic criteria for 10 PDs developed by comparing self-reports and diagnostic interviews based on the DSM-IV and the ICD-10 systems. We applied a 101-item subscale including only the DSM-IV related questions. The general prerequisite criteria for a diagnosis were confirmed by a five-item ‘Impairment and Distress Scale’ addressing interpersonal and major daily life problems caused by the individual’s personality (5 = *distress and reduced functioning*, 0 = *no problems*). The DIP-Q was validated in the Swedish population in 1998 [44] and has been included in several Scandinavian studies [43, 45–47]. Earlier publications indicate acceptable agreement at the DSM-IV cluster level (Cohen’s κ 0.45–0.63) with an overall sensitivity of 0.84, and specificity of 0.77. The self-report vs. interview correlations of dimensional scores for each personality disorder cluster were moderately high: ICC 0.60 to 0.78 [44, 46].

Using Cronbach’s α when investigating the reliability of a scale with dichotomous variables is not recommended, since it tends to underestimate the reliability scores of such scales [48]. We therefore performed confirmatory factor analysis and calculated composite reliability (CR) of the DIP-Q subscales [49]. The CFA is presented in a supplementary file (Additional file 1). We observed acceptable CR for avoidant (CR = 0.89), narcissistic (CR = 0.87), schizotypal (CR = 0.90), schizoid (CR = 0.78), paranoid (CR = 0.89), antisocial (CR = 0.78), borderline (CR = 0.85), dependent (CR = 0.71), histrionic (CR = 0.78) and obsessive-compulsive (CR = 0.65) PDs.

Dimensional classification of PDs seems to provide a better understanding of relations between diagnostic entities and their relations to maternal behaviour [24]. We therefore applied the DIP-Q subscales dimensionally in our analysis.

##### Maternal depressive symptoms

*Beck Depression Inventory (BDI–II)* [50]. The BDI is a self-report instrument covering 21 issues with four statements of increasing severity, each describing the situation over the past 2 weeks. The statements are scored from 0 to 3 and the interpretation of the total score is as follows: 0–13, no indication of depression; 14–19, mild depressive symptoms; 20–28, moderate depressive symptoms; 29–63, severe depressive symptoms. The scale has been thoroughly validated and is widely used in clinical practice [51, 52]. Cronbach’s α was 0.88 in the current study.

#### Assessment at one-year follow-up (T2)

##### Mother–child interaction observation

*Emotional Availability Scales (EAS)* [23]. Based on the theoretical work of Robert Emde [53] and attachment theory, the EAS is a research-based method for understanding the quality of communication and bidirectional emotional exchange in mother–child interactions. The scales comprise six dimensions. The adult dimensions are 1) *adult sensitivity* (i.e., a variety of adult qualities that keep the mother warm and emotionally connected to the child: responsiveness, congruence and synchronicity as well as effective conflict-solving strategies), 2) *adult structuring* (i.e., the adult’s ability to follow the child’s lead and to set limits in a firm manner, creating a scaffolding for the interaction as a ‘secure base’ and a ‘responsible adult’), 3) *adult non-intrusiveness* (i.e., absence of tendencies towards over-directiveness, over-stimulation, interference or over-protectiveness), and 4) *adult non-hostility* (i.e., absence of observed hostility, both overt and covert). The child dimensions include 5) *child responsiveness* (i.e., emotional regulation and organisation of affect/behaviour, adequate responsiveness, age-appropriateness, autonomy seeking, physical positioning and lack of role-reversal/avoidance/exclusion of the adult) and 6) *child involvement of the adult* (simple/elaborate initiative, use of the adult, lack of over-involvement, adequate eye contact/verbal involvement and body positioning). Each dimension comprises seven indicators that are assessed on either a three- or a seven-point Likert scale, representing the accurately observed capacity of both the adult and the child in the interaction. The minimum and maximum scores for the EAS subscales used in the current study are 7 and 29 points, respectively. High scores indicate good emotional availability in the dyad. The method has been validated [22, 54–56].

The video recordings were scored by four coders who were trained and certificated by Zeynep Biringen in how to administer the fourth edition of the EAS. All raters were blinded to other information regarding the family that had been filmed.

Cronbach’s α for the total EAS score was 0.97. Intra-class correlations (ICC) were used to analyse the inter-rater agreement for the EAS subscales. In the mixed-effects model, the total variance is the sum of three variance components: variance between individuals, variance between raters and residual variance [57]. The ICC was calculated to be: 0.58 (adult sensitivity), 0.53 (adult structuring), 0.50 (adult intrusiveness), 0.81 (adult hostility), 0.36 (child responsiveness) and 0.50 (child involvement). Pearson correlations were: 0.65 (adult sensitivity), 0.35 (adult structuring), 0.76 (adult hostility), 1.00 (adult intrusiveness), 0.63 (child responsiveness) and 0.64 (child involvement).

### Putative moderators/confounders

Our sample was selected from an RCT of the effect of a video-feedback intervention [41]. We have therefore investigated whether the intervention moderated the effect of maternal PDs on mother–toddler interactions.

Since the original study revealed that depressive symptoms moderated the treatment effect of the intervention, we correspondingly adjusted for this possible moderator effect as well as the intervention effects in the present study. Evidence for associations between PDs and depressive symptoms is well-established [58]. The participating mothers reported higher depressive symptoms at inclusion than at follow-up; we therefore performed secondary analyses that adjusted for the baseline depression-score (T1) to control for the possible effect on mother-toddler interaction at follow up.

Furthermore, the associations between parental PDs and parent-child interactions seem to vary with the child’s developmental stage [29]. Hence, we controlled for child age in the analyses.

### Statistics

We performed regression analyses with each of the mother–toddler interaction subscales as dependent variables: maternal sensitivity, maternal structuring, maternal non-intrusiveness, maternal non-hostility, toddler’s responsiveness and toddler’s involvement. We carried out separate analyses with each of the 10 PD symptom scales as covariates. First, these analyses were carried out unadjusted. Second, we adjusted for treatment group (TG), maternal depression at T1 (BDI), their interaction (TG × BDI), and child age. Third, we included adjustments for the interaction between the PD symptom category and treatment group for the PD symptoms where we found significant effects (for instance; TG × avoidant PD).

A two-sided *p*-value < 0.05 was selected to indicate statistical significance. Because of multiple hypotheses, *p*-values between 0.01 and 0.05 should be interpreted with caution. Ninety-five per cent confidence intervals (CI) are reported where relevant. The CFA was carried out in Mplus; all other analyses were conducted in SPSS 20.

#### Extent of missing data

Because 34 BDI forms were missing (22%), the actual number of questionnaires included in the analysis was 118. A total of 122 cases had complete or partially missing values for some items on the 101-item version of the DIP-Q questionnaire. Data was missing for 11 (10.8%) of the variables and 32 (26.2%) of the cases. However, only 256 (2.1%) of the 102 × 122 = 12,444 data values were missing. These were singly imputed using the expectation-maximation (EM) algorithm, with the 102 variables as predictors. Afterwards, values outside the limits 1–2 were set to the appropriate limit.

There were two cases with missing values for all EAS items.

#### Interrater reliability of EAS scores

The interrater reliability of the EAS scores was analysed as follows: 36 distinct individuals were selected at random, 12 from each of the three time points in the intervention study from which our sample was selected (i.e., from baseline, after the intervention and at the 6-month follow-up) (see Additional file 2) [41]. Each individual was assessed by two raters from a pool of four raters. All six combinations of raters assessed two individuals at each of the three time points. To calculate the ICC, we used a mixed-effects model with the time point (1, 2, 3) as the categorical covariate (also called the fixed factor) and with the individual and the rater as crossed random factors. With this analysis, we could examine whether some raters tended to give consistently higher scores than others. In addition, we calculated Pearson’s correlation coefficient for each of the six pairs of raters; each pair had six combinations of individuals and time points rated and then averaged these six coefficients.

## Results

Generally, our sample reported low frequencies of symptoms of PDs, ranging from 0.41 to 3.88 symptoms per disorder, with the highest number of symptoms for obsessive-compulsive, avoidant and borderline PD (where 56.6, 21.3 and 15.6% of the sample reported symptoms over the cut-off values for a possible diagnosis, respectively) (Table 2). Of the 122 women, 49 (40.2%) scored below the cut-off level for any diagnosis, while 36 (29.4%), 21 (17.2%), 9 (7.4%), 3 (2.5%), 0 and 4 (3.3%) scored over the cut-off level for one to six diagnoses, respectively. However, only 16.4% reported symptoms over the cut-off level for a putative diagnosis on the Impairment and Distress Scale (Table 2). The parents with the highest depression and personality disorder symptoms tended to participate in follow-ups less frequently (Table 2).

### Maternal PD symptom associations with maternal interactions

In the regression analyses, mothers with schizotypal PD symptoms (*n* = 10 or 8.2% with symptoms over cut-off for a putative diagnosis) showed significantly less sensitivity (β = − 0.82, *p* = 0.002), structuring (β = − 0.58, *p* = 0.002) and lower levels of non-intrusiveness (β = − 0.85, *p* = 0.004) in the interaction with their toddler (Table 3). Adjusting for treatment group (TG) and depressive symptoms at T1 (BDI), the interaction between treatment group and depression (TG × BDI) and child age did not change the β- or the *p*-values substantially (β = − 0.92 to − 0.98, *p*-values = 0.001 for maternal sensitivity; β = − 0.65 to − 0.72, *p*-values = 0.006 to 0.008 for maternal structuring; β = − 0.91 to − 1.01, *p*-values = 0.001 to 0.003 for maternal non-hostility) (see Table 4). Adjusting for all the mentioned confounders/moderator in treatment group versus control group resulted in higher *p*-values, relative to the low frequencies of schizotypal PD symptoms and multiple adjustments. Even if the moderating effects of treatment group did not turn out to be statistically significant (see the section ‘Moderator analysis’), schizotypal mothers in the intervention group tended to be more sensitive and less hostile, but at the same time were less structured and more intrusive in the interaction with their toddlers compared to control mothers (Table 5).

**Table 3:** Associations between specific maternal PD symptoms and EAS subscales, unadjusted

Covariates	Maternal sensitivity					Maternal structuring				
Personality disorder symptoms	β	CI	*p*-value	R^2^	*n*	β	CI	*p*-value	R^2^	*n*
Avoidant	− 0.11	− 0.55 to 0.34	0.64	< 0.01	94	−0.03	− 0.42 to 0.36	0.87	< 0.01	94
Dependent	−0.20	− 0.70 to 0.30	0.42	0.01	94	− 0.25	− 0.69 to 0.19	0.21	0.01	94
Obsessive-compulsive	0.00	−0.48 to 0.48	0.99	< 0.01	94	0.00	−0.43 to 0.43	1.00	< 0.01	94
Paranoid	−0.26	−0.84 to 0.33	0.39	0.01	94	− 0.25	−0.77 to 0.27	0.38	0.01	94
Schizoid	−0.43	− 1.24 to 0.38	0.30	0.01	94	− 0.24	− 0.94 to 0.47	0.50	0.01	94
Schizotypal	**− 0.82**	**− 1.32 to − 0.32**	**0.002**	**0.10**	94	**− 0.58**	**− 1.03 to − 0.12**	**0.01**	**0.07**	94
Antisocial	− 0.18	− 1.05 to 0.69	0.68	< 0.01	94	− 0.05	− 0.82 to 0.72	0.90	< 0.01	94
Borderline	**− 0.50**	**− 0.96 to − 0.03**	**0.04**	**0.05**	94	−0.29	− 0.71 to 1.24	0.17	0.02	94
Histrionic	0.41	−0.25 to 1.06	0.22	0.02	94	0.42	−0.16 to 0.99	0.15	0.02	94
Narcissistic	−0.89	−1.81 to 0.03	0.06	0.04	94	−0.53	−1.35 to 0.29	0.20	0.02	94
	Maternal non-hostility					Maternal non-intrusiveness				
Avoidant	−0.70	−0.36 to 0.22	0.63	< 0.01	94	0.03	−0.46 to 0.52	0.90	0.08	94
Dependent	−0.05	−0.38 to 0.27	0.74	< 0.01	94	−0.01	− 0.56 to 0.55	0.98	< 0.01	94
Obsessive-compulsive	− 0.05	− 0.36 to 0.26	0.76	< 0.01	94	− 0.02	− 0.55 to 0.52	0.96	< 0.01	94
Paranoid	−0.02	− 0.40 to 0.36	0.93	< 0.00	94	−0.29	− 0.94 to 0.36	0.37	0.01	94
Schizoid	−0.21	− 0.73 to 0.32	0.44	0.01	94	− 0.09	−1.0 to 0.82	0.84	< 0.01	94
Schizotypal	−0.31	−0.61 to 0.02	0.07	0.04	94	**− 0.85**	**− 1.41 to 0.28**	**0.004**	**0.09**	94
Antisocial	−0.40	−0.96 to 0.16	0.16	0.02	94	− 0.26	− 1.23 to 0.71	0.60	< 0.01	94
Borderline	**−0.44**	**− 0.73 to 0.14**	**0.004**	**0.09**	94	− 0.31	− 0.83 to 0.22	0.25	0.01	94
Histrionic	0.11	−0.32 to 0.54	0.61	< 0.01	94	0.46	−0.26 to 1.19	0.21	0.02	94
Narcissistic	−0.10	−0.71 to 0.51	0.74	< 0.01	94	− 0.69	− 0.73 to 0.35	0.19	0.02	94
	Child responsiveness					Child involvement				
Avoidant	−0.05	− 0.48 to 0.39	0.83	< 0.01	94	0.05	−0.48 to 0.58	0.84	< 0.01	94
Dependent	−0.13	−0.62 to 0.37	0.62	< 0.01	94	−0.24	−0.34 to 0.80	0.43	0.01	94
Obsessive-compulsive	0.12	−0.36 to 0.59	0.63	< 0.01	94	0.23	−0.83 to 0.35	0.43	0.01	94
Paranoid	−0.46	−1.03 to 0.11	0.11	0.03	94	−0.26	−0.96 to 0.44	0.46	0.01	94
Schizoid	−0.39	−1.19 to 0.42	0.34	0.01	94	−0.51	−1.47 to 0.46	0.30	0.01	94
Schizotypal	**−0.71**	**−1.22 to 0.21**	**0.006**	**0.08**	94	−0.49	−1.12 to 1.113	0.12	0.03	94
Antisocial	−0.10	− 0.96 to 0.76	0.82	< 0.01	94	0.00	−1.04 to 1.04	1.00	< 0.01	94
Borderline	−0.31	− 0.74 to 0.13	0.16	0.02	94	− 0.20	− 0.73 to 0.32	0.45	0.01	94
Histrionic	0.48	−0.16 to 1.13	0.14	0.02	94	**0.89**	**0.12 to 1.65**	**0.02**	**0.06**	94
Narcissistic	**−1.04**	**−1.94 to − 0.13**	**0.03**	**0.05**	94	− 1.01	−2.11 to 0.09	0.07	0.04	94

Regression coefficients (unstandardised β, 95% CI, p-value and adjusted R^2^) of the ten categories of personality disorder symptoms, one at the time as covariates, and the EAS adult subscales as dependent variables. Significant findings are shown bold

**Table 4:** Associations between maternal schizotypal PD symptoms and adult EAS subscales with adjustments

Covariates	Maternal sensitivity					Maternal structuring				
Personality disorder symptoms	β	CI	*p*-value	R^2a^	*n*	β	CI	*p*-value	R^2^	*n*
Unadjusted										
Schizotypal	**− 0.82**	**−1.32 to − 0.32**	**0.002**	**0.10**	94	**−0.58**	**−1.03 to − 0.12**	**0.01**	**0.07**	94
Adjusted separately for										
Child age	**−0.92**	**−1.45 to − 0.39**	**0.001**	**0.12**	89	**−0.65**	**−1.13 to − 0.17**	**0.008**	**0.09**	89
BDI (T1)	**−0.98**	**−1.53 to − 0.43**	**0.001**	**0.10**	89	**−0.71**	**− 0.20 to − 0.21**	**0.006**	**0.06**	89
Treatment group (TG)	**−0.95**	**−1.48 to − 0.42**	**0.001**	**0.13**	89	**−0.68**	**−1.17 to − 0.20**	**0.006**	**0.07**	89
BDI, TG, TG × BDI	**−0.96**	**− 0.51 to − 0.41**	**0.001**	**0.11**	89	**− 0.71**	**−1.21 to − 0.21**	**0.006**	**0.05**	89
Adjusted for all in										
Treatment group^b^	**−0.87**	**−1.63 to − 0.11**	**0.03**	**0.10**	89	**−0.84**	**−1.52 to − 0.15**	**0.02**	**0.06**	89
Control group^c^	**−1.03**	**− 1.84 to − 0.21**	**0.01**	**0.10**	89	**−**0.52	− 1.26 to − 0.11	0.16	0.06	89
	Maternal non-hostility					Maternal non-intrusiveness				
Personality disorder symptoms	β	CI	*p*-value	R^2^	n	β	CI	*p*-value	R^2^	n
Unadjusted										
Schizotypal	−0.31	−0.61 to 0.02	0.07	0.04	94	**−0.85**	**− 1.41 to 0.28**	**0.004**	**0.09**	94
Adjusted separately for										
Child age	−0.28	− 0.59 to 0.07	0.12	0.08	89	**−0.91**	**−1.51 to − 0.37**	**0.003**	**0.09**	89
BDI (T1)	−0.32	−0.69 to 0.05	0.09	0.01	89	**−1.01**	**−1.63 to − 0.40**	**0.002**	**0.09**	89
Treatment group (TG)	−0.31	−0.67 to 0.05	0.09	0.02	89	**−0.96**	**−1.55 to − 1.36**	**0.002**	**0.08**	89
BDI, TG, TG × BDI	−0.31	−1.68 to 0.07	0.11	< 0.01	89	**− 1.02**	**− 1.64 to − 0.41**	**0.001**	**0.09**	89
Adjusted for all in										
Treatment group^b^	−0.10	− 0.60 to 0.41	0.71	0.06	89	**−1.01**	**− 1.95 to 0.24**	**0.01**	**0.08**	89
Control group^c^	−0.51	−1.04 to 0.03	0.07	0.06	89	**−0.91**	**−1.82 to 0.01**	**0.05**	**0.08**	89

Significant findings are shown bold

^a^Adjusted R^2^

^b^(adjusting for schizotypal PD × Treatment group in the original RCT study)

^c^(adjusting for schizotypal PD × Control group in the original RCT study)

**Table 5:** Associations between maternal borderline PD symptoms and adult EAS subscales with adjustments

Covariates	Maternal sensitivity					Maternal structuring				
Personality disorder symptoms	β	CI	*p*-value	R^2a^	*n*	β	CI	*p*-value	R^2^	*n*
Unadjusted										
Borderline	**−0.50**	**− 0.96 to − 0.03**	**0.04**	**0.05**	94	**−** 0.29	−0.71 to 1.24	0.17	0.02	94
Adjusted separately for										
Child age	−0.41	−0.87 to 0.04	0.08	0.04	89	−0.27	−0.68 to 0.13	0.19	0.04	89
BDI (T1)	−0.43	−0.91 to 0.06	0.08	0.03	89	−0.30	−0.73 to 0.13	0.17	< 0.01	89
Treatment group (TG)	−0.41	−0.87 to 0.06	0.09	0.03	89	−0.28	−0.69 to 0.69	0.19	< 0.01	89
BDI, TG, TG × BDI	−0.40	−0.89 to 0.09	0.11	0.01	89	−0.30	−0.74 to 0.15	0.19	− 0.02	89
Adjusted for all in										
Treatment group^b^	−0.67	− 1.44 to 0.10	0.09	0.01	89	−0.51	−1.20 to 0.17	0.14	< 0.01	89
Control group^c^	−0.22	− 0.86 to 0.42	0.50	0.01	89	−0.15	− 0.72 to 0.42	0.61	< 0.01	89
	Maternal non-hostility					Maternal non-intrusiveness				
Personality disorder symptoms	β	CI	*p*-value	R^2^	*n*	β	CI	*p*-value	R^2^	*n*
Unadjusted										
Borderline	**−0.44**	**−0.73 to 0.14**	**0.004**	**0.09**	94	−0.31	**−**0.38 to 0.22	0.25	0.01	94
Adjusted separately for										
Child age	**−0.37**	**−0.65 to − 0.09**	**0.01**	**0.07**	89	−0.21	− 0.73 to 0.31	0.42	0.01	89
BDI (T1)	**−0.40**	**−0.71 to − 0.09**	**0.01**	**0.07**	89	−0.28	− 0.82 to 0.26	0.31	0.01	89
Treatment group (TG)	**−0.38**	**−0.67 to − 0.08**	**0.01**	**0.07**	89	−0.21	− 0.73 to 0.31	0.42	0.01	89
BDI, TG, TG × BDI	**−0.39**	**− 0.70 to − 0.09**	**0.01**	**0.05**	89	− 0.27	−0.81 to 0.27	0.32	0.01	89
Adjusted for all in										
Treatment group^b^	−0.40	− 0.84 to 0.12	0.14	0.09	89	−0.51	−1.38 to 0.35	0.24	0.02	89
Control group^c^	**−0.43**	**− 0.83 to − 0.04**	**0.01**	**0.09**	89	− 0.14	−0.86 to 0.58	0.28	0.02	89

Significant findings are shown bold

^a^Adjusted R^2^

^b^(adjusting for borderline PD × Treatment group in the original RCT study)

^c^(adjusting for borderline PD × Control group in the original RCT study)

Mothers with borderline PD (*n* = 19 or 15.6% with symptoms over cut-off for a putative diagnosis) were observed as less non-hostile in their interactions with their toddlers (β = − 0.44, *p* = 0.004), but the tendency to show less sensitivity to their toddlers’ signals was only marginally significant (β = − 0.50, *p* = 0.04) (Table 3). Adjusting for TG, BDI, TG × BDI and child age when investigating the effect of borderline PDs’ effect on maternal sensitivity revealed decreased β-values (Table 5). This was especially the case when we adjusted for all in the control group (β = − 0.22), where the associations were no longer significant. The effect on maternal non-hostility showed relatively unchanged β-values (− 0.37 to − 0.43) and was still highly significant when adjusting for the same variables (*p* = 0.01). When we adjusted for all in the treatment group, however, the associations were no longer significant. Hence, mothers’ borderline PDs was associated with higher levels of maternal hostility in the interaction with their toddlers.

**Fig. 1:**
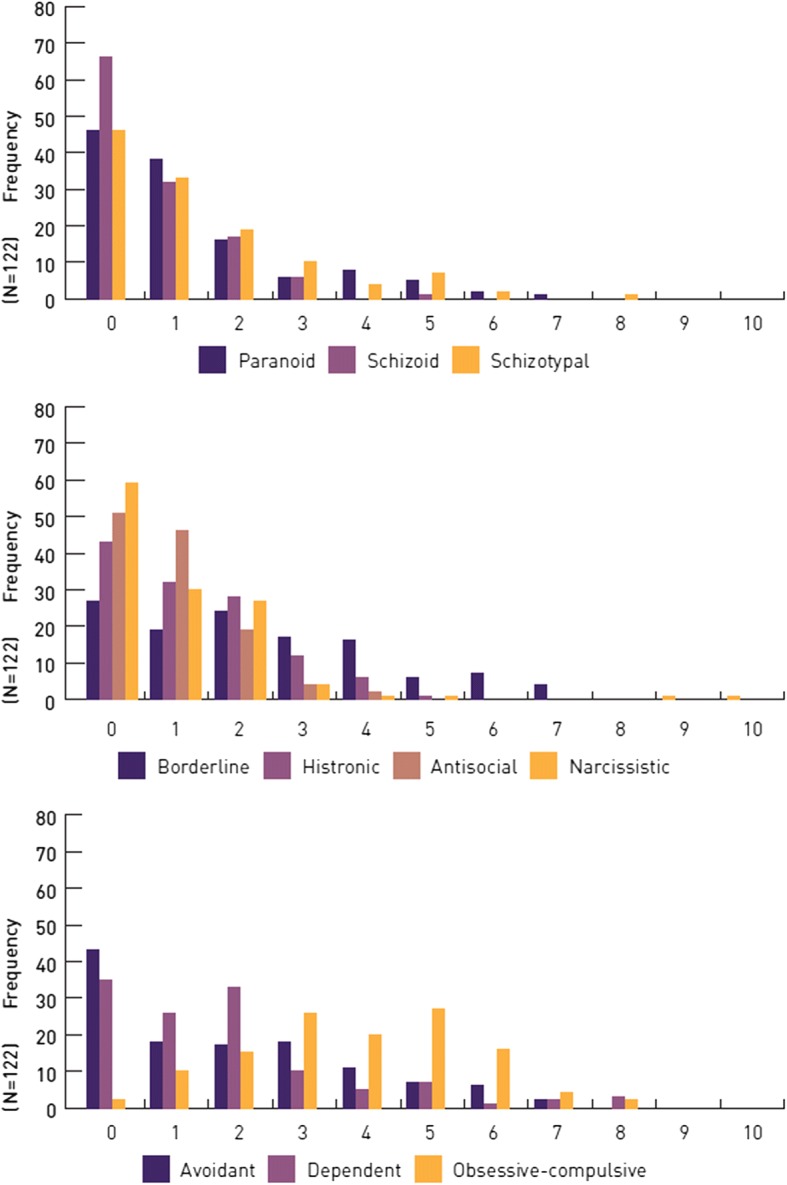
Sample frequencies (Y-axis) of confirmed symptoms (X-axis) of the ten personality disorders (PDs)

The distribution of frequencies of the different PD symptoms are presented in Fig. 1 and Table 2.

### Maternal PD symptom associations with the toddler’s interactions

Mothers with either narcissistic (*n* = 1 or 0.8% with symptoms over cut-off for a putative diagnosis) or schizotypal PD symptoms had toddlers who interacted less responsively with them (narcissistic PD, not adjusted, β = − 1.04, *p* = 0.03; schizotypal PD, not adjusted, β = − 0.71, *p* = 0.006) (Table 3). These effects remained when we adjusted for TG, BDI, TG × BDI and child age (narcissistic PD, adjusted, β = − 1.68 to − 1.09, *p* = 0.02; schizotypal PD, adjusted, β = − 0.75 to − 0.82, *p* = 0.004 to 0.007) (Table 6). When adjusting for all, the effect of maternal narcissistic symptoms on toddler’s responsiveness was only significant in the control group. Conversely, the effect of schizotypal PD on child responsiveness was only significant in the intervention group (Table 6). However, there was no large difference between β-values within the two groups, which means that this result should be interpreted with caution.

**Table 6:** Associations between maternal PD symptoms and child EAS subscales with adjustments

Covariates	Child responsiveness					Child involvement				
Personality disorder symptoms	β	CI	*p*-value	R^2a^	*n*	β	CI	*p*-value	R^2^	*n*
Unadjusted										
Schizotypal	**−0.71**	**−1.22 to 0.21**	**0.006**	**0.08**	94	−0.49	−1.12 to 1.113	0.12	0.03	94
Adjusted separately for										
BDI (T1)	**−0.83**	**−1.37 to − 0.29**	**0.003**	**0.08**	91	−0.61	−1.29 to 0.08	0.08	0.01	91
Child age	**−0.66**	**−1.17 to − 0.15**	**0.01**	**0.08**	92	−0.44	−1.08 to 0.19	0.17	0.02	92
Treatment group	**−0.72**	**−0.22 to − 0.21**	**0.006**	**0.06**	94	−0.50	−1.13 to 0.12	0.12	0.01	94
Treatment group × BDI (T1)	**−0.81**	**−1.34 to − 0.28**	**0.003**	**0.08**	91	−0.58	−1.26 to 0.09	0.09	0.01	91
Adjusted for all in										
Treatment group^b^	**−0.85**	**−1.61 to − 0.09**	**0.03**	**0.05**	89	−0.32	− 0.66 to 0.51	0.51	− 0.01	89
Control group^c^	−0.74	−1.56 to 0.08	0.08	0.05	89	−0.89	−1.92 to 0.15	0.09	−0.01	89
Unadjusted										
Narcissistic	**−1.04**	**− 1.94 to − 0.13**	**0.03**	**0.05**	94	− 1.01	− 2.11 to 0.09	0.07	0.04	94
Adjusted separately for										
BDI (T1)	**−1.10**	**− 2.02 to − 0.18**	**0.02**	**0.06**	89	−1.06	−2.20 to 0.08	0.07	0.04	89
Child age	**−1.12**	**−2.03 to − 0.21**	**0.02**	**0.07**	89	−1.09	−2.22 to 0.04	0.06	0.06	89
Treatment group	**−1.09**	**−2.01 to − 0.17**	**0.02**	**0.06**	89	−1.07	−2.21 to 0.07	0.06	0.06	89
Treatment group × BDI (T1)	**−1.10**	**−2.02 to − 0.18**	**0.02**	**0.06**	89	−1.06	**−**2.20 to 0.08	0.07	0.04	89
Adjusted for all in										
Treatment group^b^	**−**0.94	**−**2.80 to 0.93	0.32	0.04	89	−1.52	−3.83 to 0.80	0.20	0.02	89
Control group^c^	**−1.68**	**−3.06 to − 0.30**	**0.02**	**0.04**	89	**−2.00**	**−3.69 to − 0.20**	**0.02**	0.02	89
Unadjusted										
Histrionic	0.48	−0.16 to 1.13	0.14	0.02	94	**0.89**	**0.12 to 1.65**	**0.02**	**0.06**	94
Adjusted separately for										
BDI (T1)	0.45	−0.27 to 1.08	0.24	0.02	89	**0.84**	**0.03 to 1.66**	**0.04**	**0.05**	89
Child age	0.40	−0.22 to 1.12	0.19	0.02	89	**0.89**	**0.09 to 1.70**	**0.03**	**0.05**	89
Treatment group	0.40	−0.27 to 1.08	0.24	0.02	89	**0.83**	**0.02 to 1.65**	**0.05**	**0.05**	89
Treatment group × BDI (T1)	0.40	−0.28 to 1.07	0.25	0.02	89	**0.84**	**0.03 to 1.66**	**0.04**	**0.05**	89
Adjusted for all in										
Treatment group^b^	0.13	− 0.94 to 1.20	0.81	< 0.01	89	0.59	−0.71 to 1.89	0.72	0.01	89
Control group^c^	0.66	− 0.26 to 1.57	0.16	0.02	89	1.05	**−**0.06 to 2.15	0.06	0.04	89

Significant findings are shown bold

^a^Adjusted R^2^

^b^(adjusting for schizotypal PD × Treatment group in the original RCT study)

^c^(adjusting for schizotypal PD × Control group in the original RCT study)

Finally, mothers with schizotypal PD symptoms in the intervention group tended to have more involved toddlers compared to control mothers. However, the moderator analysis showed no statistically significant effect of schizotypal PDs on the toddler’s interactions (see next section).

Maternal histrionic PD traits were associated with more involved children (β = 0.89, *p* = 0.02). When adjusting for TG, BDI, TG × BDI and child age, the β-values remained relatively unchanged, but the *p*-values tended to be higher or the associations were no longer statistically significant (Table 6). Thus, mothers with narcissistic and schizotypal PD symptoms seem to have less responsive toddlers.

### Moderator analysis

As a last step in our analysis, we investigated the possible moderating effect of the intervention from the original RCT from which our sample was selected. Except for the families in which mothers reported paranoid and dependent PD symptoms, the intervention group had no influence on the associations between maternal PDs and maternal sensitivity, structuring, non-hostility, non-intrusiveness or toddler’s responsiveness and involvement (*p*-values between 0.08 and 0.98). For mothers with paranoid PD symptoms (*n* = 8 or 6.6% with symptoms over cut-off for a putative diagnosis), we observed that the intervention group significantly influenced the associations between symptoms of paranoid PD and maternal structuring (*p* = 0.002), non-intrusiveness (*p* = 0.002) as well as toddler’s involvement (*p* = 0.002) and responsiveness (*p* = 0.004). Therefore, we performed a secondary analysis where we explored the associations between paranoid PD symptoms and mother– toddler interaction adjusting for the moderating effect, paranoid PD × TG, and for all the covariates (TG, BDI, TG × BDI and child age). With the new adjustments, we found significant associations between paranoid PD symptoms and maternal structuring (β = − 1.34, *p* = 0.03, CI = − 2.58 to − 0.11), maternal non-intrusiveness (β = − 1.83, *p* = 0.02, CI = − 3.38 to − 0.28), and toddlers’ responsiveness (β = − 1.54, p = 0.03, CI = − 2.91 to − 0.16).

We also observed that the intervention group significantly influenced the associations between symptoms of dependent PD (*n* = 13 or 10.7% with symptoms over cut-off for a putative diagnosis) and maternal non-hostility (p = 0.02). Because we conducted 60 moderator analyses (10 PD categories × 6 outcomes × 1 moderator), it is quite plausible that these results are spurious findings and they were therefore not emphasised in our discussion or conclusions.

### Maternal comorbidity

As depicted in Table 7, mothers’ symptoms of schizotypal PD correlated with symptoms of all the other PDs as well as maternal depressive symptoms, while symptoms of borderline PD correlated with symptoms of all other PDs except for schizoid. Furthermore, for mothers displaying symptoms over the cut-off level for a diagnosis for either borderline (*n* = 19, 15.6%) or schizotypal PD (*n* = 10, 8.2%), the mean symptoms frequencies were over the cut-off levels for one or two other PDs, respectively (Table 8). The mean scores of depression symptoms were in the mild to moderate range among those who also had symptoms over the cut-off scores for either schizotypal or borderline PDs. Hence, we observed an accumulative comorbidity risk with increasing PD symptoms. The sample size was too small to allow us to adjust for symptoms of all other PDs.

**Table 7:** Pearson correlations between symptoms of the ten PDs and maternal depression

PD symptoms/depressive symptoms	1	2	3	4	5	6	7	8	9	10	11
1 Avoidant		**0.61** ^**^	**0.46** ^**^	**0.53** ^**^	**0.18** ^*^	**0.45** ^**^	0.04	**0.45** ^**^	0.11	0.16	**0.58** ^**^
2 Dependent			**0.35** ^**^	**0.41** ^**^	0.17	**0.35** ^**^	0.11	**0.44** ^**^	0.15	0.17	**0.63** ^**^
3 Obsess. Comp.				**0.26** ^**^	0.12	**0.24** ^**^	−0.08	**0.28** ^**^	0.07	0.15	**0.43** ^**^
4 Paranoid					**0.38** ^**^	**0.51** ^**^	0.11	**0.40** ^**^	0.14	**0.19** ^*^	**0.36** ^**^
5 Schizoid						**0.28** ^**^	0.07	0.17	0.07	**0.31** ^**^	**0.20** ^*^
6 Schizotypal							**0.30** ^**^	**0.61** ^**^	**0.28** ^**^	**0.31** ^**^	**0.34** ^**^
7 Antisocial								**0.40** ^**^	**0.40** ^**^	0.16	−0.06
8 Borderline									**0.50** ^**^	**0.37** ^**^	**0.41** ^**^
9 Histrionic										0.18	0.07
10 Narcissistic											0.12
11 BDI total score											

* *p* < 0.05, ** *p* < 0.01. Significant findings are shown bold

**Table 8:** Descriptive statistics of the subgroups of Schizotypal/Borderline PD

Schizotypal PD symptoms ≥5				Borderline PD symptoms ≥5			
PD symptoms	*n*	mean	sd	PD symptoms	*n*	mean	sd
Dependent	10	3.60	1.71	Dependent	19	3.63	2.63
Obsessive-comp.	10	**4.50**	1.08	Obsessive-comp.	19	**4.74**	1.15
Avoidant	10	3.80	2.20	Avoidant	19	3.79	2.12
Paranoid	10	3.60	2.41	Paranoid	19	2.63	2.29
Schizoid	10	1.40	1.65	Schizoid	19	1.16	1.07
Antisocial	10	1.60	1.17	Schizotypal	19	3.11	1.82
Borderline	10	**5.60**	2.67	Antisocial	19	1.63	0.76
Histrionic	10	1.90	1.28	Histrionic	19	2.47	1.39
Narcissistic	10	2.10	1.37	Narcissistic	19	1.74	1.41
BDI mean score	9	19.89	6.83	BDI mean score	17	18.64	11.33

Mothers with symptoms over the cut-off score of a diagnosis. Bold numbers mean that the mean values are higher than the cut-off value for a diagnosis of the respective PD

## Discussion

Using a longitudinal design, we examined how self-reported PD symptoms were associated with mothers’ sensitivity and structuring ability and their intrusiveness and hostility in interactions with their toddler a year after initial assessment. Furthermore, we examined the relationship between the toddler’s responsiveness or involvement towards their mothers and maternal PD symptoms.

### Levels of impairment and distress

We observed a low mean level of impairment and distress in our sample (Table 2). However, 16.4% of the mothers reported symptoms over the cut-off value on the impairment and distress scale, which is regarded as the single most important predictor of concurrent or prospective dysfunction in association with PDs [13]. This indicates slightly elevated subjective experienced morbidity related to their interpersonal functioning compared to representative non-clinical samples; the prevalence of any PD range between 11 and 14% in other Scandinavian studies including non-clinical samples, while the prevalence in clinical samples is 59% [46, 47, 59]. The family interaction problems might primarily have been limited to the mother–toddler relationship or were possibly attributed to the child. Hence, the mothers might not have experienced or acknowledged the same extent of problems in socialising with others. The mothers actually reported high degrees of satisfaction with their network support and low levels of conflict with others (Table 1). On the other hand, 63.8% of the participants were still on maternity leave (49–59 weeks in Norway) at the time they were interviewed (Table 1). Thus, the low degree of distress reported by most mothers may have been coloured by a less stressful family situation.

### Mothers with schizotypal PD symptoms and characteristic interactions with their toddlers

We observed that mothers with schizotypal PD symptoms were less sensitive and had lower capacity for structuring in the interactions. Clearly, schizotypal mothers’ lower interactional competence in the mother–toddler relationship may derive from constricted affect, which is a diagnostic criterion for schizotypal PD. According to the EAS, less sensitivity indicates less joy, less creativity, and decreased mutual emotional exchange between the mother and the child. It might also indicate the display of inappropriate adult affects in the relationship, as well as a decreased ability to handle conflict in an appropriate manner. The aloofness or decreased ability to ‘fit in’ in a smooth, socially accepted manner, as described in persons with schizotypal symptoms in the DSM-IV, may be characteristic of these mothers’ reduced sensitivity in interactions with their toddlers. The observed lower abilities for structuring also indicate that these mothers use less guidance and suggestions regarding the toddler’s play, set fewer limits or boundaries or have low success rates when doing so. Because odd, vague or incoherent speech is regarded as a central symptom in individuals with schizotypal PD, mothers with these traits may struggle to use clear language when guiding their toddlers in the acquisition of new skills or solving problems. To the best of our knowledge, this is the first study to find that mothers with schizotypal PD symptoms are less sensitive and structuring in their interactions with their toddlers.

Moreover, mothers with symptoms of schizotypal PD were observed to be more intrusive in interactions with their toddlers. One may suspect a decreased ability to follow the child’s lead or a tendency to interrupt, indicating that the mothers were less aware of their toddler’s signals or the importance of timing when responding to the child’s initiatives. Our findings support earlier research suggesting that schizotypal individuals are somewhat egocentric, with a reduced cognitive capacity to read other people’s intentions [60].

Maternal psychosis was an exclusion criterion for participation in this study. However, phenomena of influence, derealisation (i.e., an alteration in the experience or perception of the external world) and magical thinking were reported surprisingly frequently within our sample (see Additional file 3). Even when they have insufficient information to make plausible inferences, schizotypal individuals nevertheless seek to explain what occurs around them, often resulting in faulty interpretations and magical thinking [60]. It is likely such misinterpretations also occur with their children, potentially causing the observed interactional problems.

Currently, researchers include schizotypal PD among the schizophrenic spectrum disorders because the conditions are genetically linked and show both neuro-anatomical and physiological similarities to schizophrenia [61–63]. Few individuals in our sample had symptoms that would have met the diagnostic criteria for the schizophrenia-related spectrum disorder or schizotypal PD, especially in view of the low prevalence of schizotypal PD (0.6%) in Norway [59]. The lifetime incidence of schizophrenia is also low (0.3–0.7%) [64]. When these facts and the low levels of impairment and distress are considered, it is therefore noteworthy that even the very few schizotypal PD symptoms in our sample predicted poorer maternal and child interactional capacities.

### Characteristics of toddlers in interactions with mothers with schizotypal PD symptoms

The toddlers of mothers with schizotypal PD symptoms were no less involved in interactions with their mothers than toddlers of mothers without such symptoms. These toddlers might not expect their mothers to be ‘adequately emotionally present’ and may therefore assume an active role in the interaction. However, when the mothers initiated contact towards them, the toddlers were significantly less responsive, which indicates lower emotional availability towards the mother [23]. Despite involving the mother instrumentally on a behavioural level, the children displayed an emotional shutdown state with an over-regulation of emotions as a consequence of decreased maternal ability to engage in a reciprocal exchange.

### Characteristics of mother–child interactions when mothers report borderline PD symptoms

We observed that mothers with symptoms of borderline PD were more hostile in their interactions with their toddlers, even in this non-clinical sample. Our study also revealed that mothers with symptoms of borderline PD had a tendency to be less sensitive in interactions with their children. However, since multiple analyses were performed, this result must be interpreted with caution. We did not observe less responsive or involved children.

The mothers’ higher hostility scores represent a state of negativity, anger, covert or overt hostility, or disrespectfulness, or show that the mothers were not able to maintain composure during stress. Nonetheless, one could speculate as to why these toddlers were observed as being responsive, which meant that they showed clear signs of pleasure, eagerness or willingness in the interaction with their mothers, who were observed as being marginally sensitive or inconsistent in their behaviours. Child involvement was also observed as normal in our sample. It might be possible that these mothers showed behaviours when they were not video-taped, which instigated the child’s involvement [65, 66]. Rutter and Quinton [67] observed that having a mother with a PD characterised by high levels of hostility was a greater risk factor for poor mental health in the child than having a mother with schizophrenia or bipolar disorder. Therefore, children’s efforts to initiate involvement with their mothers and be overly responsive towards them when faced with maternal hostility and marginal levels of sensitivity, might represent an unhealthy coping strategy on the part of the child [68–70].

Individuals with borderline PD symptoms are well-known for their emotional instability. However, because the mothers could choose when and in which activities they were to be filmed whilst interacting with their toddlers, this may have rendered it more difficult for the coders to detect their instability, meaning that only marginally significant negative associations between borderline symptoms and maternal sensitivity were observed. Mothers with subclinical levels of borderline PD symptoms are, most likely, able to display adequate emotional availability, stable affect and appropriate behaviour when being filmed on their own terms, at least for a limited amount of time (30 min in our study). Filming multiple situations could have revealed a tendency towards shifting/contradictory behaviours or impulsive affective communication often associated with mothers with borderline PD [35, 36].

### Associations between mother–child interactions and other maternal PD symptoms

We observed that paranoid mothers were less structuring and more intrusive in the interaction with their toddlers. Furthermore, paranoid and narcissistic traits in the mothers predicted reduced child responsiveness, while histrionic traits predicted increased child involvement. Because multiple analyses were conducted, these results must also be interpreted with caution. To the best of our knowledge, only one other study has investigated the connections between maternal symptoms of paranoid and narcissistic PD and maternal over-involvement or child responsiveness/involvement in mother–child interactions [29]. In that study, no significant findings were reported on the connections between narcissistic symptoms and child involvement. However, paranoid mothers showed increased tendencies to control their children, while children of paranoid mothers were reported as being more compliant.

### Clinical implications

To recognise mothers with ‘low threshold’ schizotypal PD symptoms in clinical practice may be difficult because such patients may appear withdrawn and anxious, particularly with regard to social interactions in unfamiliar settings, and hence they may be less likely to reveal their problems. Furthermore, increased tenseness and suspicion towards others in mothers with schizotypal PD symptoms may complicate attempts to discuss the mother–child relationship at a well-baby unit. A thorough routine examination of the mother’s relationship with their child in the early visits during the postpartum period may, however, reveal a pattern of relationship difficulties that could serve as a trigger for appropriate actions at an early stage (such as more frequent home visits by a well-baby nurse or a parenting intervention). The short video-based intervention offered to families in the treatment group instigated tendencies to increased sensitivity and less hostility in maternal behaviours and more involved children in the parent–toddler interactions. Since a general stability of schizotypal personality disorders symptoms is expected over time [71], these families should probably be offered more extensive and lengthy treatment.

Even in a non-clinical sample, mothers’ borderline PD symptoms appeared to be problematic because these mothers exhibited higher levels of hostility towards their children. To recognise these mothers might be even more challenging, especially if the children remain responsive and involved towards their mothers and if the mothers are able to ‘behave as expected’. Signs of maternal covert hostility and child role reversal might be indicators of an unhealthy condition.

Treatment of family interaction difficulties and parents’ psychopathology are usually anchored in segregated professional disciplines that have limited knowledge of each other’s fields of expertise [14]. Several interventions have been found to be effective in treatment of family interaction problems (see overviews; [72, 73]). Except for the documentation that exists on the treatment of borderline PDs, less evidence on the effect of treatment of other adult PDs exists [74, 75]. Even fewer studies have investigated the effects of interventions targeting dually disordered mother–child dyads where the mothers are suffering from PDs [71, 76]. This study has therefore attempted to bridge the gap between these fields of clinical practice by quantifying the associations between parents’ PD symptoms and their relational problems with their small children. It is, however, important to replicate these findings in further research, including non-clinical samples. It is especially imperative that future research evaluate the effects of an integrated intervention model targeting dually disordered mother–child dyads for mothers with symptoms of schizotypal and borderline PDs to prevent unhealthy developmental trajectories for the children.

### Methodological issues

The ICC of the EAS subscales ranged between 0.35 and 0.81, which would be considered low in some contexts. However, it should be noted that this low ICC is caused by the relatively large residual variances compared to the between-individual variances. The inter-rater variances were by far the smallest of these variance components, so the contribution to the total variance from inter-rater variance is practically negligible.

The average Pearson correlation between the raters ranged from low to high (0.35 to 1.00). The Pearson correlation coefficient and the variance components from the mixed model address different issues. For instance, if one rater consistently rated scores exactly 20 points higher than another rater, the Pearson correlation between the two would be 1.0. On the other hand, the relative magnitude of the inter-rater variance in the mixed model tells us that there were no large systematic differences between the raters.

To the best of our knowledge, there was no potential bias interfering with our analysis. However, we tried to lower the risk of bias by defining relatively rigorous inclusion- and exclusion criteria, using validated measure methods, and using a standardised, blind data collection method. Furthermore, we selected families living in both rural and urban parts of the country and chose a prospective design to reduce the risk of selection bias.

## Conclusions

Mothers’ schizotypal personality symptoms appear to compromise maternal sensitivity and structuring as well as increasing tendencies of intrusiveness in mother–child interactions. Moreover, mothers’ schizotypal symptoms were associated with less responsive toddlers in the mother–child relationship.

Mothers’ borderline PD symptoms were associated with higher levels of hostility in the mothers’ interactions with their toddler, although the mothers were only marginally less sensitive.

### Limitations

The histories of earlier maternal psychiatric disorders that may have interfered with the mothers’ capacity for self-observation and with how the mothers responded to self-report questionnaires were not obtained in the current inquiry, and neither were records of ongoing medication acquired.

In both epidemiological and clinical studies, comorbidity among individuals with PDs is common [46, 77, 78]; thus our findings represent a ‘true’ picture. When interpreting our results, the total comorbidity must be kept in mind as it predicts a large impact on how the mothers interact with their child. Other possible confounding variables might also be involved in the transfer of child risk related to mothers’ PDs; in particular, the severity of mothers’ PDs [7, 29, 33, 45, 46, 79, 80]. We adjusted for child age, maternal depressive symptoms, treatment group and their interactions. However, since stratification on PD disorders is not recommended and the sample was size restricted, we did not adjust for other confounders. Generally, research in the area is limited, and the exploration of other confounding variables is correspondingly sparse. Potentially, a variety of early life stressors and genetic liabilities are also involved in the transmission of child developmental risk related to maternal PDs.

## Additional files

Additional file 1: Results from the confirmatory factor analyses of the DIP-Q subscales in MPlus. Model fit coefficients for the ten personality disorder subscales. (DOC 23 kb)

Additional file 2: Enrolment in the original RCT study versus current study. Flow chart of the recruitment process of the effect study of a parenting intervention, which the present paper is based on. (DOC 34 kb)

Additional file 3: Frequencies of responses on the different DIP-Q items confirming schizotypal PD symptoms (*n* = 122). Item content and frequencies of responses of the schizotypal personality disorder subscale. (DOC 29 kb)

## Abbreviations

BDI: Beck Depression Inventory
DIP-Q: DSM-IV and ICD-10 Personality Questionnaire
EAS: Emotional Availability Scales
PD: Personality disorder
RCT: Randomised controlled trial
