# Correction to: Maternal personality disorder symptoms in primary health care: associations with mother–toddler interactions at one-year follow-up

**DOI:** 10.1186/s12888-018-1962-x

**Published:** 2018-12-13

**Authors:** Magnhild Singstad Høivik, Stian Lydersen, Ingunn Ranøyen, Turid Suzanne Berg-Nielsen

**Affiliations:** 1 Department of Mental Health, Faculty of Medicine and Health Sciences, the Norwegian University of Science and Technology (NTNU), N-7491 Trondheim, Norway; 2 Regional Centre for Child and Youth Mental Health and Child Welfare – Central Norway, Faculty of Medicine and Health Sciences, The Norwegian University of Science and Technology (NTNU), Trondheim, Norway; 3 Division of Psychiatry, St Olavs Hospital, Trondheim University Hospital, Trondheim, Norway; 4 The Centre for Child and Adolescent Mental Health, Eastern and Southern Norway, Oslo, Norway

**Correction to: BMC Psychiatry (2018) 18:198**

**DOI: 10.1186/s12888-018-1789-5**

Following publication of the original article [1], the authors notified us of an error in the data presented in Table 2: the DIP-Q subscales show an incorrect scale.

The corrected Table 2 is presented below.

**Table 2:** Sample clinical characteristics at baseline and at one-year follow-up

Baseline										Follow up					
	All participants			Attrition group			Remaining group								
Personality disorder symptoms	n	mean	sd	n	mean	sd	n	mean	sd	n	mean	sd	cut off (diagnosis)	n (%) with symptoms over cut off	Scale range
Avoidant	122	1.93	1.95	28	2.29	2.56	94	1.82	1.85	–	–	–	≥ 4	26 (21.3)	0–7
Dependent	122	1.81	1.88	28	**2.61** ^***^	2.39	94	**1.57** ^***^	1.64	–	–	–	≥ 5	13 (10.7)	0–9
Obsessive-compulsive	122	3.88	1.74	28	4.00	1.89	94	3.84	1.70	–	–	–	≥ 4	69 (56.6)	0–9
Paranoid	122	1.34	1.60	28	**2.04** ^***^	2.03	94	**1.14** ^***^	1.40	–	–	–	≥ 4	8 (6.6)	0–8
Schizoid	122	0.73	0.97	28	0.68	0.86	94	0.75	1.00	–	–	–	≥ 4	1 (0.8)	0–8
Schizotypal	122	0.41	1.66	28	2.04	1.88	94	1.22	1.55	–	–	–	≥ 5	10 (8.2)	0–11
Antisocial	122	0.85	0.85	28	0.86	0.85	94	0.85	0.94	–	–	–	≥ 3	6 (4.9)	0–10
Borderline	122	2.48	2.15	28	**3.61** ^***^	2.69	94	**2.14** ^***^	1.85	–	–	–	≥ 5	19 (15.6)	0–17
Histrionic	122	1.25	1.22	28	1.39	1.17	94	1.21	1.24	–	–	–	≥ 5	1 (0.8)	0–8
Narcissistic	122	0.86	1.05	28	**1.29** ^**^	1.33	94	**0.73** ^**^	0.87	–	–	–	≥ 5	1 (0.8)	0–10
Impairment and distress	122	0.66	1.03	28	**0.80** ^*^	1.26	94	**0.63** ^*^	0.96	–	–	–	≥ 2	20 (16.4)	0–5
Depressive symptoms	118	12.11	8.64	24	**15.96** ^*^	11.23	94	**10.99** ^**^	7.84	85	**8.74** ^***^	7.05	–	–	0–63
EAS subscales															
Maternal sensitivity	152	22.41	5.12	42	21.48	4.94	110	22.77	5.16	110	**25.29** ^***^	3.92	–	–	7–29
Maternal structuring	152	23.26	4.50	42	22.38	4.29	110	23.60	4.55	110	**25.90** ^***^	3.39	–	–	7–29
Maternal non-hostility	152	26.01	3.58	42	**24.92** ^*^	4.18	110	**26.44** ^*^	3.24	110	**27.32** ^***^	2.50	–	–	7–29
Maternal non-intrusiveness	152	22.24	5.72	42	21.26	6.16	110	22.63	5.52	110	**25.27** ^***^	4.34	–	–	7–29
Child responsiveness	152	22.66	5.36	42	21.59	5.06	110	23.07	5.43	110	**25.70** ^***^	4.08	–	–	7–29
Child involvement	152	22.43	5.94	42	20.50	5.97	110	21.79	5.92	110	**25.11** ^***^	4.71	–	–	7–29

Maternal personality disorder symptoms measured with the DIP-Q (DSM IV and ICD-10 Personality Questionnaire)

^*^ = *p* < 0.05, ^**^ = *p* < 0.01, ^***^ = *p* < 0.001 (independent samples t-tests of characteristics in attrition compared to remaining groups in baseline sample, and paired sample t-tests comparing characteristics of remaining and follow up groups)
