# Supplementary material for: Maternal personality disorder symptoms in primary health care: associations with mother–toddler interactions at one-year follow-up
Source: BMC Psychiatry. 2018 Jun 18;18:198. doi: 10.1186/s12888-018-1789-5 (PMC6006703; doi:10.1186/s12888-018-1789-5)
Supplement: Supplementary file 1 — Results from the confirmatory factor analyses of the DIP-Q subscales in MPlus. Model fit coefficients for the ten personality disorder subscales. (DOC 23 kb) [file 12888_2018_1789_MOESM1_ESM.doc]

**Additional file 1:** Results from the confirmatory factor analyses of the DIP-Q subscales in MPlus

We observed acceptable model fits for avoidant (7 items) (CFI = 0.99, TLI = 0.99, RMSEA = 0.026), narcissistic (10 items) (CFI = 0.98, TLI = 0.98, RMSEA = 0.055), schizotypal (11 items) (CFI = 0.95, TLI = 0.94, RMSEA = 0.050), schizoid (8 items) (CFI = 0.98, TLI = 0.97, RMSEA = 0.035), paranoid (8 items) (CFI = 1.00, TLI = 1.00, RMSEA < 0.000) and antisocial (6 of 10 items) (CFI = 1.00, TLI = 1.00, RMSEA < 0.000) PDs. Less acceptable model fits were observed for borderline (15 of 16 items) (CFI = 0.89, TLI = 0.86, RMSEA = 0.118,), dependent (9 items) (CFI = 0.87, TLI = 0.83, RMSEA = 0.082), histrionic (8 items) (CFI = 0.61, TLI = 0.45, RMSEA = 0.160) and obsessive-compulsive (8 of 9 items) (CFI = 0.73, TLI = 0.62, RMSEA = 0.082) PDs, indicating more than one latent construct. Six questions were removed from the subscales because they had negative loadings on the latent constructs: Question 26 (an obsessive-compulsive item), Questions 69, 71, 72 and 74 (antisocial items) and Question 92 (a borderline item).
