# Supplementary material for: Maternal personality disorder symptoms in primary health care: associations with mother–toddler interactions at one-year follow-up
Source: BMC Psychiatry. 2018 Jun 18;18:198. doi: 10.1186/s12888-018-1789-5 (PMC6006703; doi:10.1186/s12888-018-1789-5)
Supplement: Supplementary file 2 — Enrolment in the original RCT study versus current study. Flow chart of the recruitment process of the effect study of a parenting intervention, which the present paper is based on. (DOC 34 kb) [file 12888_2018_1789_MOESM2_ESM.doc]

**Additional file 2: Enrolment in the original RCT study versus current study**

**T1 current study**

**n=152**

**T2 current study**

**n=110**

Intervention 4-5 months

Assessed for eligibility (n=180)

Excluded (n=22)

♦  Not meeting inclusion criteria (n=15 )

♦  Declined to participate/

withdrew (n=7)

T3 original study

**♦ Had a follow up evaluation six months after intervention (n=65)**

T2 original study

♦ Had a post treatment evaluation (n=73)

T1 original study

Allocated to intervention (n=88)

**♦ Had a pre-treatment evaluation (n= 86)**

T2 original study

♦ Had a post treatment evaluation (n=52)

T1 original study

Allocated to intervention (n=70)

♦ **Had a pre-treatment evaluation (n= 66)**

T3 original study

**♦ Had a follow up evaluation six months after intervention (n=45)**

Randomized (n=158)
