# Supplementary material for: Maternal personality disorder symptoms in primary health care: associations with mother–toddler interactions at one-year follow-up
Source: BMC Psychiatry. 2018 Jun 18;18:198. doi: 10.1186/s12888-018-1789-5 (PMC6006703; doi:10.1186/s12888-018-1789-5)
Supplement: Supplementary file 3 — Frequencies of responses on the different DIP-Q items confirming schizotypal PD symptoms (n = 122). Item content and frequencies of responses of the schizotypal personality disorder subscale. (DOC 29 kb) [file 12888_2018_1789_MOESM3_ESM.doc]

**Additional file 3: Frequencies of responses on the different DIP-Q items confirming schizotypal PD symptoms (n = 122)**

| ***DIP-Q Item*** | ***True / Partially true (%)*** | ***Not true (%)*** |
| --- | --- | --- |
| “*I often think that people are talking behind my back.*”  “*I often pick up hidden meanings in what people say or do.*”  “*I can communicate with others by means of telepathy.*”  “*I have a sixth sense for knowing when things will happen before they actually do.*”  “*I often mistake objects and shadows in a room for human figures.*”  “*I often have strange bodily experiences that others have difficulty in understanding.*”  “*People normally think I express myself in a strange way.*”  “*Other people react to my way of expressing feelings.*”  “*Most people probably think that I am odd, eccentric or peculiar.*”  “*I feel comfortable when I am with people I know.*”  “*There are those who think that I am emotionally cold and have a negative manner.*” | 13.93 / 1.64  22.10  3.30 / 1.60  12.30 / 1.60  4.92 1.64  5.74 / 0.82  11.48 / 0.82  5.74 / 0.82  27.90  97.54 / 1.64  32.80 | 84.43  77.90  95.10  86.10  93.44  93.44  87.70  93.44  72.10  0.82  67.20 |
